# Natural variability of lung function in primary ciliary dyskinesia: longitudinal analysis from the PROVALF-PCD cohort

**DOI:** 10.1183/23120541.01115-2024

**Published:** 2025-06-23

**Authors:** Kewei Zhang, Avni Kant, Mieke Boon, Melissa Borrelli, Carolina Constant, Silvia Castillo Corullon, Renato Cutrera, Stefanie Dillenhöfer, Sanem Eryılmaz Polat, Ela Eralp, Nathalie Feyaerts, Amanda Harris, Claire Hogg, Cordula Koerner-Rettberg, Panayiotis Kouis, Enrico Lombardi, Natalie Lorent, June K. Marthin, Vendula Martinu, Antonio Moreno-Galdo, Lucy Morgan, Kim Nielsen, Heymut Omran, Ugur Ozcelik, Petr Pohunek, Phil Robinson, Sandra Rovira-Amigo, Francesca Santamaria, Anne Schlegtendal, Aline Tamalet, Guillaume Thouvenin, Dilber Ademhan Tural, Nicola Ullmann, Woolf T. Walker, Panayiotis Yiallouros, Camille Pearse, Bettina Frauchiger, Claudia E. Kuehni, Nicole Beydon, Philipp Latzin, Jane S. Lucas, Bruna Rubbo

**Affiliations:** 1 Clinical and Experimental Sciences, University of Southampton Faculty of Medicine, Southampton, UK; 2 Primary Ciliary Dyskinesia Centre, University Hospital Southampton NHS Foundation Trust, Southampton, UK; 3 Department of Respiratory Diseases, University Hospitals Leuven, Leuven, Belgium; 4 Department of CHROMETA, Laboratory for Respiratory Diseases and Thoracic Surgery, KU Leuven, Leuven, Belgium; 5 Department of Translational Medical Sciences, Pediatric Pulmonology, Federico II University, Naples, Italy; 6 Department of Pediatrics, Hospital de Santa Maria, Centro Hospitalar Universitário Lisboa Norte and Faculty of Medicine, University of Lisbon, Lisbon, Portugal; 7 FE Pediatría y Neumología Infantil, Unidad de Neumología Infantil y Fibrosis Quística, Hospital Clínico de Valencia, Valencia, Spain; 8 Respiratory and Cystic Fibrosis Unit, Academic Pediatric Department, Bambino Gesù Children Hospital, IRCCS, Rome, Italy; 9 Department of Paediatric Pulmonology, University Children's Hospital, Ruhr University Bochum, Katholisches Klinikum Bochum, Bochum, Germany; 10 Hacettepe University Faculty of Medicine, Department of Pediatric Pulmonology, Ankara, Turkey; 11 Marmara Universitesi, Department of Pediatric Pulmonology, Istanbul, Turkey; 12 Royal Brompton and Harefield NHS Foundation Trust, Department of Paediatrics, Primary Ciliary Dyskinesia Centre, London, UK; 13 Children's Hospital and Research Institute, Marienhospital Wesel, Wesel, Germany; 14 Medical School, University of Cyprus, Nicosia, Cyprus; 15 Meyer Children's Hospital IRCCS, Florence, Italy; 16 Danish PCD and chILD Centre, CF Centre Copenhagen, Paediatric Pulmonary Service, ERN Accredited, Department of Paediatrics and Adolescent Medicine, Copenhagen University Hospital, Rigshospitalet, Copenhagen, Denmark; 17 Department of Clinical Medicine, University of Copenhagen, Copenhagen, Denmark; 18 Department of Paediatrics, Charles University, 2nd Faculty of Medicine, University Hospital Motol, Prague, Czech Republic; 19 Paediatric Pulmonology Section, Department of Paediatrics, Hospital Universitari Vall dHebron, Barcelona, Spain; 20 Universitat Autònoma de Barcelona, Instituto de Salud Carlos III, Centre for Biomedical Network Research on Rare Diseases (CIBERER), Barcelona, Spain; 21 Concord Hospital, Concord Clinical School, University of Sydney, Department of Respiratory Medicine, Sydney, Australia; 22 Department of General Pediatrics, University Hospital Muenster, Muenster, Germany; 23 Faculty of Medicine Dentistry and Health Sciences, Paediatrics, University of Melbourne, Melbourne, Australia; 24 Sorbonne Université, Inserm U938, Centre de Recherche Saint-Antoine, Paris, France; 25 Sorbonne Université, Assistance Publique – Hôpitaux de Paris, Hôpital Trousseau, Service de Pneumologie Pédiatrique, Paris, France; 26 MRC Lifecourse Epidemiology Centre, University of Southampton, Southampton, UK; 27 Division of Paediatric Respiratory Medicine and Allergology, Department of Paediatrics, Inselspital, University Hospital, University of Bern, Bern, Switzerland; 28 Institute of Social and Preventive Medicine, University of Bern, Bern, Switzerland; 29 AP-HP, Sorbonne Université, Unité d'Exploration Fonctionnelle Respiratoire, Hôpital Armand-Trousseau, and Sorbonne Université, INSERM U938, Paris, France; 30 These authors contributed equally

## Abstract

**Background:**

The extent to which changes in lung function are due to natural variability in patients with primary ciliary dyskinesia (PCD) is unknown. We aimed to assess intra-individual variability in forced expiratory volume in 1 s (FEV_1_) derived from spirometry to define the extent to which the observed changes were due to test variability in clinically stable PCD patients.

**Methods:**

PROVALF-PCD (Prospective Observational Multicentre Study on Variability of Lung Function in Stable PCD Patients) was a large international prospective cohort conducted in 2017–2019. We included patients aged ≥5 years who were clinically stable at two or more consecutive visits and provided spirometry-derived lung function measurements. To calculate the upper limit of normal (ULN), we fitted an unadjusted multilevel mixed-effect model, and to determine the absolute change in FEV_1_ z-scores, we calculated the coefficient of repeatability (CR). We performed sensitivity analyses by stratifying relative change by age (adults *versus* children), number of measurements (at least four), and time between measurements (<4 months apart).

**Results:**

We included 252 participants from 12 countries with confirmed or highly likely PCD. We included 1028 FEV_1_ measurements from patients in stable state. The ULN for relative change between two measurements of FEV_1_ was 25%. Test variability remained high in all sensitivity analyses. The CR was 1.88 FEV_1_ z-score.

**Conclusions:**

Changes in intra-individual FEV_1_ >25% between visits in stable PCD patients lie beyond the expected test variability and therefore could be considered physiologically relevant. These findings inform the selection of end-points for pulmonary intervention trials in PCD, as they suggest that FEV_1_ is not a sensitive test for monitoring lung health in PCD.

## Shareable abstract

**This large international longitudinal study found high intra-individual variability in FEV_1_ z-scores from consecutive visits in stable patients with primary ciliary dyskinesia. Findings should inform selection of primary outcomes for future clinical trials.** https://bit.ly/3PaIeJe

## Introduction

Spirometry is frequently used to monitor disease progression and response to treatment in those with lung disease. Spirometry is generally recommended every 3–6 months in individuals with primary ciliary dyskinesia (PCD), with results compared to reference data and previous measurements from the same patient [1, 2]. For meaningful interpretation of whether a change in lung function is likely clinically relevant, we need to understand the extent of intra-individual variability between clinic measurements, in addition to the normal age-dependent increase in absolute lung function during childhood and the normal age-dependent decline in adulthood. Previous studies in healthy children assessing within-individual variability have shown a between-measures variability mean±sd of 0.05±0.6 z-scores, with 95% of all the children achieving a between-measure variability within ±1.3 z-scores over the course of 1 year [3].

Determining whether a measured change reflects a physiologically meaningful alteration or test variability can be challenging, and this distinction is important in both clinical and research settings [4, 5]. Anecdotally, there is significant variability within individuals between measures in forced expiratory volume in 1 s (FEV_1_) derived from spirometry in stable adults and children with PCD, perhaps due to the volume and consistency of mucus secretions, even when adhering to standards for measurement, analysis and reporting, and when using reference equations (*e.g.* Global Lung Function Initiative (GLI)) [6]. However, there are limited longitudinal data on FEV_1_ changes in patients with PCD [7–9]. Furthermore, questions have been raised over the sensitivity of FEV_1_ to monitor disease progression for people with PCD, particularly its ability to detect early deterioration of lung function compared to other methods, such as the lung clearance index (LCI) from multiple breath washouts (MBW) [10, 11]. Additionally, FEV_1_ derived from spirometry is being used as a primary end-point in clinical trials assessing the efficacy of drugs in improving lung function or preventing the progression of lung disease in PCD patients [12, 13].

We aimed to prospectively investigate intra-individual variations in the lung function parameter FEV_1_ between consecutive spirometry measurements, to define the extent to which the observed changes were due to expected test variability in clinically stable patients with PCD.

## Methods

### Study population and design

PROVALF-PCD (Prospective Observational Multicentre Study on Variability of Lung Function in Stable PCD Patients) is a large, international cohort initiated by the Better Experimental Approaches to Treat PCD (BEAT-PCD) network [14–17]. The inclusion criteria were 1) confirmed or highly likely diagnosis of PCD according to European Respiratory Society guidelines [18, 19]; 2) age ≥5 years; 3) clinically stable (*i.e.* not experiencing a clinician-defined pulmonary exacerbation at the time of the clinical appointment) in at least two consecutive appointments; and 4) had at least two consecutive appointments ≤6 months apart. We excluded those where the interval between routine spirometry measurements was >6 months, or where the diagnostic status was inconclusive (*i.e.* did not have sufficient test results to be classified as either “PCD highly unlikely” or “PCD highly likely”).

Information on patients’ demographics, clinical history, diagnostics tests, appointment data and spirometry measurements were collected during routine clinical visits every 3–6 months between August 2017 and December 2019. Participants should have had a minimum of two clinical appointments during the study period, but could have had up to 10. Pseudo-anonymised data were captured using a study-specific case record form. They were entered by each participating centre into a dedicated online database, available through Research Electronic Data Capture (REDCap). REDCap is a secure, web-based software platform designed to support data capture for research studies, with audit trails for tracking data management [20, 21]. Centres only had access to their own data. Additional information on the PROVALF-PCD cohort has been published elsewhere [22].

### Ethics

This study was approved by the Ethics and Research Governance Online service provided by the University of Southampton (Southampton, UK) (ID: ERGO 27420), under more comprehensive approval for the collection of clinical data for use in research (National Research Ethics Service Committee South Central Hampshire A Ethics 06/Q1702/109).

Appropriate ethics, informed consent and other approvals were obtained locally by each centre. Centres provided a signed paper-based or scanned copy of the agreement, to be kept at the University of Southampton. Data were pseudonymised locally before transfer to the University of Southampton for analysis, adhering to international data transfer agreements signed by all parties. The study protocol was registered at ClinicalTrials.gov (identifier NCT03704896).

### Study outcome

The outcome variable was FEV_1_ derived from spirometry, obtained during clinical visits from patients in stable state. Stable state was defined as not experiencing an episode of pulmonary exacerbation when attending a clinical appointment. Presence of pulmonary exacerbation was assessed and determined by the clinician(s) responsible for the patient's clinical care, during the routine clinical appointment. Spirometry data obtained from patients experiencing a clinician-defined pulmonary exacerbation were excluded from the analyses.

Lung function measurements were carried out by qualified technicians and clinicians at each centre during routine clinical appointments, using their clinic's equipment. Centres were provided with a study-specific standard operation procedure document, adapted from Miller *et al.* [23], to ensure that measurements and data collection were standardised. The document contained detailed information on equipment use and calibration, test procedures and quality control (supplementary material).

We calculated FEV_1_ z-scores using the GLI 2012 references [24], which are adjusted for sex, age, height and ethnicity (determined by clinicians or self-declared by the participant) on the day of the lung function test. We calculated FVC z-scores using the same method (results in the supplementary material).

### Study covariates

Body mass index (BMI) z-scores were calculated based on the bodyweight and height collected at clinical visits, using the World Health Organization references [25]. As there are no international references for those aged >19 years, values for adults were calculated based on the assumption that they were aged 19 years.

Microbiology cultures were prospectively collected during routine clinical appointments and tested at each centre's microbiology laboratory. Type of respiratory tract sample and presence (or absence) of each pathogen were recorded. The health score was comprised of the participant's perceived self-reported health compared to their usual health, and was classified as very well, well, somewhat well, ill, or very ill.

We defined adult participants as those aged ≥18 years and children as those aged <18 years.

### Statistical methods

We described continuous variables as median and interquartile range (IQR), and categorical variables as total numbers and proportions. We calculated relative change in FEV_1_ between consecutive visits and the upper limit of normal (ULN) as detailed in the supplementary material. We fitted an unadjusted multilevel model with random intercept at patient level to calculate the ULN for relative change, as detailed in the supplementary material.

To investigate the determinants of individual variability of lung function, we investigated the association between ULN for relative change of FEV_1_ and sex, ultrastructural defect by transmission electron microscopy (TEM), presence of respiratory pathogens and FEV_1_ at baseline in multivariate models. To investigate the contribution by each covariate, we ran univariate models, stratified by children and adults, and to assess whether an initially low or high FEV_1_ z-score would impact our findings, we also adjusted these models by FEV_1_ at baseline. We reported the estimates (β-coefficients), with 95% confidence intervals, and the adjusted R^2^.

We constructed unadjusted multilevel models with random intercepts at patient and country levels to estimate the absolute change of FEV_1_ z-scores between consecutive visits. We also calculated the coefficient of repeatability (CR) as detailed in the supplementary material. The CR is used to determine short-term variability or test measurement noise, where changes that lie outside the test's CR parameter can be considered beyond test variability [26].

To investigate long-term trends for lung function progression, we constructed multilevel linear models with random intercepts at patient and country level, and FEV_1_ z-score values as outcome. We fitted unadjusted and adjusted models (adjusted for the following covariates: time since initial appointment, BMI z-scores, presence of respiratory pathogens, health score, number of antibiotics used since the last visit, use of antibiotic prophylaxis, and use of inhaled corticosteroid at the time of their clinical appointment), with FEV_1_ z-scores from measurements obtained from patients in stable state as outcome. We fitted the same model with relative change in FEV_1_ as outcome. Intra-class correlation coefficient (ICC) was calculated based on these models, to assess the proportion of the overall variation explained by clustering within people and within country. The latter can be used to indicate whether there are considerable differences between measurements obtained from different countries.

We conducted four sensitivity analyses. To determine the extent of intra-individual variability in children and adults separately, we stratified the relative and absolute change in FEV_1_ by children and adults. To assess the impact of having more than two measurements of FEV_1_ per participant, we conducted two subgroup analyses: 1) including only those who had at least three measurements and 2) including only those who had at least four measurements during the follow-up period. Lastly, we restricted our analyses to only those who had between-measurement intervals of <4 months, since test variability is higher when time between measurements increases [4, 6].

Data coding, cleaning, analyses and plotting were conducted in Rstudio version 4.4.0, using the broom.mixed, jtools, lme4 and tidyverse packages.

## Results

We analysed data from 252 participants from 19 PCD centres in 12 countries (supplementary table S1). There were 1178 visits in which lung function was assessed between August 2017 and December 2019 (median (IQR) 3 (2–4) visits per participant) (supplementary table S1), of which 419 measurements were from 84 adults and 759 measurements were from 168 children. Of those, 1028 (87.3%) measurements were taken when the participant was in stable state (*i.e.* not experiencing an episode of pulmonary exacerbation). All measurements obtained during an episode of pulmonary exacerbation were excluded from our analyses.

Median (IQR) age at recruitment was 14.7 (11.2–19.8) years (table 1). Median (IQR) FEV_1_ z-score at baseline was −1.66 (−2.74–0.48). Longitudinal trends for FEV_1_ z-score varied between countries, but there were no clear distinct patterns or larger variations in any given country (supplementary figure S1). Most lower airway microbiology samples were from sputum (92%), and a respiratory pathogen was isolated in 62.6% of samples (supplementary table S2).

**TABLE 1:** Characteristics of participants included in the study

**Subjects**	252 (100)
**Visits**	1178 (100)
Stable visits	1028 (87.3)
**Female**	117 (46.0)
**Age at baseline years**	14.7 (11.2–19.8)
**Age at diagnosis years**	6 (1.1–11.5)
**BMI at baseline z-score**	0.12 (−0.68–0.85)
**FEV_1_ at baseline z-score**	−1.66 (−2.74– −0.48)
**FEV_1_ at baseline L**	2.24 (1.7–2.9)
**Ethnicity**	
White	233 (92.5)
Black	3 (1.2)
North-East Asian	0 (0)
South-East Asian	1 (0.4)
Other/mixed	15 (6.0)
**Cardiac situs**	
Situs solitus	131 (52.0)
Situs inversus	113 (44.8)
Situs ambiguous	7 (2.8)
Unknown	1 (0.4)

Data are presented as n (%) or median interquartile range. BMI: body mass index; FEV_1_: forced expiratory volume in 1 s.

Of the 252 participants, 181 (72%) were classified as confirmed PCD based on TEM and/or genetics, and the others were highly likely PCD based on clinical history, nasal nitric oxide testing and high-speed video analysis [18]. There were 125 (50%) participants with abnormal ciliary ultrastructure detected by TEM, and biallelic mutations were identified in PCD-causative genes in 113 (45%) participants (supplementary figure S2, table 2).

**TABLE 2:** Diagnostics for the 252 patients included in the study

	All patients	Children	Adults
**Patients**	252	168	84
**TEM**			
ODA^#^	59 (23.4)	41 (24.4)	18 (21.4)
IDA and ODA^#^	45 (17.9)	29 (17.3)	16 (19.0)
Microtubular disorganisation with IDA^#^	21 (8.3)	15 (8.9)	6 (7.1)
IDA	7 (2.8)	6 (3.6)	1 (1.2)
Central pair defect	6 (2.4)	1 (0.6)	5 (6.0)
Absent/completely reduced cilia	5 (2.0)	4 (2.4)	1 (1.2)
Other	3 (1.2)	2 (1.2)	1 (1.2)
Normal TEM test	23 (9.1)	15 (8.9)	8 (9.5)
Inconclusive	19 (7.5)	16 (9.5)	3 (3.6)
Not performed	64 (25.3)	39 (23.2)	25 (29.8)
**Genetic testing**			
Positive genetic test	113 (44.8)	83 (49.4)	30 (35.7)
No genetic diagnosis found	9 (3.6)	5 (3.0)	4 (4.8)
Not performed	130 (51.6)	80 (47.6)	50 (59.5)
**Nasal nitric oxide testing**			
Low (<77 nL·min^−1^)	148 (58.7)	95 (56.5)	53 (63.1)
Normal (>77 nL·min^−1^)	13 (5.2)	11 (6.5)	2 (2.4)
Not performed	91 (36.1)	62 (36.9)	29 (34.5)
**High-speed video microscopy analysis**			
Static or static with residual movement	165 (65.5)	113 (67.3)	52 (61.9)
Rotating/circular movement	3 (1.2)	2 (1.2)	1 (1.2)
Abnormal CBP likely secondary (*e.g.* infection, mucus impedance)	3 (1.2)	3 (1.8)	0 (0)
Other abnormal CBP	6 (2.4)	3 (1.8)	3 (3.6)
Normal	1 (0.4)	0 (0)	1 (1.2)
Inconclusive	26 (10.3)	15 (8.9)	11 (13.1)
Not performed	48 (19)	32 (19.0)	16 (19.0)

Data are presented as n or n (%). TEM: transmission electron microscopy; ODA: outer dynein arm defect; IDA: inner dynein arm defect; CBP: ciliary beating pattern. ^#^: class I defects, according to Shoemark *et al.* [19].

### Relative change in FEV_1_

**FIGURE 1:**
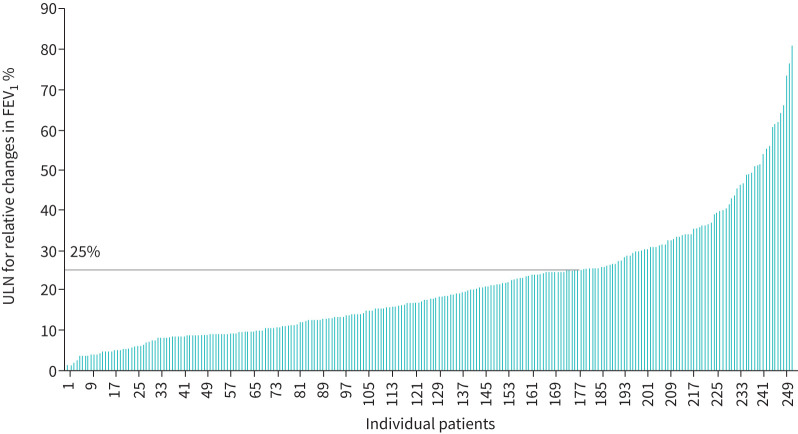
Forced expiratory volume in 1 s (FEV_1_) relative change upper limit of normal (ULN) for individuals in stable state between two consecutive clinic appointments (n=252 patients). The horizontal line shows the ULN for patients in stable status. Changes above this value would be considered physiologically relevant (*i.e.* not due to the test variability).

The ULN between two measurements of FEV_1_ obtained ≤6 months apart was 25% in stable patients. Between-measures changes in FEV_1_ for individual patients, expressed as relative change, varied between 1% and 81% (figure 1). 22 participants had a between-measures relative change >40%, of which in 15 participants the observed changes were an increase, and in seven participants, these were a decrease in FEV_1_ z-scores. In the latter, the relative change of FEV_1_ z-score decreased between 43% and 77%. The participant with the highest decrease in relative change (*i.e.* 77%) had a baseline FEV_1_ z-score of −2.54 at the age of 14 years, followed by −2.45 FEV_1_ z-score at the second appointment, and −4.47 at the third appointment, despite reportedly not having a pulmonary exacerbation.

When stratifying the analyses by children and adults, the ULN between two measurements of FEV_1_ was 20.5% in adults and 26.9% in children (p=0.77). In the sensitivity analyses, the ULN when considering only those with at least three measurements was 25%, and 24% when considering only those with at least four measurements. When restricting our analyses to participants who had measurements obtained <4 months apart, the ULN was 24%.

### Absolute change in FEV_1_ z-score

The mean absolute difference in intra-individual FEV_1_ z-score was relatively small (−0.013), but had a large standard deviation (0.68). The CR was 1.88, meaning that 95% of absolute differences in FEV_1_ z-score between measurements were expected to fall <1.88. When stratifying the analyses by children and adults, the CR for children was 2.05 and for adults was 1.58.

**FIGURE 2:**
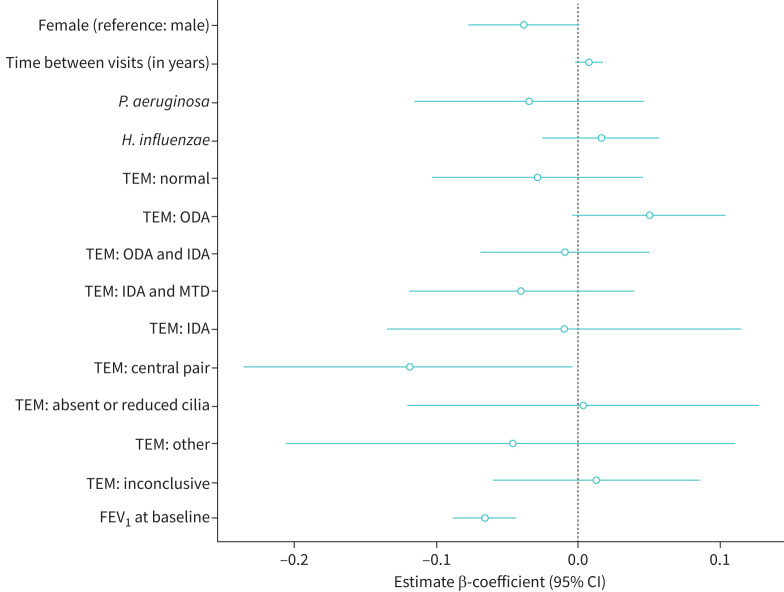
Estimates (β-coefficients) for covariates included in fully adjusted model, with individual relative variability (95% upper limit of normal) of forced expiratory volume in 1 s (FEV_1_) as outcome. Model was adjusted by all covariates shown in the figure. *P. aeruginosa*: *Pseudomonas aeruginosa*; *H. influenzae*: *Haemophilus influenzae*; TEM: transmission electron microscopy; ODA: outer dynein arm defect; IDA: inner dynein arm defect; MTD: microtubular disarrangement.

Individual relative change in FEV_1_, expressed as 95% ULN for each individual, was slightly lower in females (β-coefficient −0.04, 95% CI −0.08–0.00), in those with central pair defect by TEM (β-coefficient −0.12, 95% CI −0.23–0.00), and in those with lower FEV_1_ at baseline (β-coefficient −0.06, 95% CI −0.09– −0.04) in the fully adjusted models (figure 2). In the univariate models, participants with outer dynein arm (ODA) defect had FEV_1_ variability of 0.05 (95% CI 0.00–0.10), while those with central pair defect had variability of −0.13 (95% CI −0.26– −0.02), when adjusted for FEV_1_ values at baseline (table 3). Children with ODA defect also had slightly higher variability when adjusted for FEV_1_ values at baseline (β-coefficient 0.06, 95% CI 0.00–0.13) (table 3). The adjusted R^2^ was 0.19, which means that these factors explained <20% of the total individual variability observed. Most of the variability was explained by FEV_1_ value at baseline (adjusted R^2^ from the univariate model with FEV_1_ at baseline as single exposure=0.14).

**TABLE 3:** Determinants of individual relative variability (95% upper limit of normal) for forced expiratory volume in 1 s (FEV_1_) in 201 patients with primary ciliary dyskinesia with complete data available (141 children and 60 adults).

	All	Children	Adults	Adjusted^#^ all	Adjusted^#^ children	Adjusted^#^ adults
**Sex**						
Female (reference: male)	−0.01 (−0.05–0.03)	−0.01 (−0.06–0.04)	0 (−0.06–0.06)	−0.03 (−0.07–0)	−0.02 (−0.07–0.02)	−0.03 (−0.09–0.04)
**TEM**						
Normal	−0.01 (−0.09–0.07)	0 (−0.10–0.09)	−0.04 (−0.18–0.09)	−0.02 (−0.10–0.05)	−0.03 (−0.12–0.06)	−0.04 (−0.17–0.09)
ODA	0.05 (−0.01–0.10)	0.06 (−0.01–0.13)	0.01 (−0.08–0.10)	0.05 (0–0.10)	0.06 (0.00–0.13)	0.02 (−0.07–0.11)
ODA and IDA	−0.02 (−0.08–0.04)	−0.02 (−0.10–0.06)	−0.01 (−0.10–0.08)	−0.01 (−0.07–0.04)	−0.02 (−0.09–0.05)	−0.01 (−0.10–0.08)
IDA and MTD	−0.02 (−0.11–0.06)	−0.04 (−0.15–0.07)	0.02 (−0.10–0.13)	−0.03 (−0.10–0.05)	−0.02 (−0.12–0.08)	0.01 (−0.11–0.12)
IDA	0.03 (−0.11–0.16)	0.01 (−0.13–0.16)	NA	0.01 (−0.12–0.13)	0 (−0.14–0.13)	NA
Central pair	−0.14 (−0.26–−0.02)	−0.12 (−0.42–0.19)	−0.11 (−0.23–0.01)	−0.13 (−0.24–−0.01)	−0.09 (−0.37–0.18)	−0.11 (−0.23–0.01)
Absent or reduced cilia	0.01 (−0.12–0.15)	−0.03 (−0.19–0.13)	0.19 (−0.05–0.43)	0.01 (−0.12–0.13)	−0.06 (−0.20–0.09)	0.20 (−0.04–0.44)
Other	−0.10 (−0.27–0.07)	−0.13 (−0.35–0.09)	−0.04 (−0.27–0.20)	−0.06 (−0.22–0.10)	−0.09 (−0.29–0.11)	−0.02 (−0.26–0.21)
Inconclusive	0.03 (−0.05–0.11)	0.04 (−0.06–0.13)	−0.03 (−0.18–0.12)	0.02 (−0.05–0.09)	0.02 (−0.10–0.10)	−0.02 (−0.17–0.13)
**Presence of *P. aeruginosa***	0 (−0.08–0.09)	0 (−0.12–0.13)	0.03 (−0.07–0.14)	−0.01 (−0.09–0.07)	0 (−0.11–0.12)	0.02 (−0.09–0.12)
**Presence of *H. influenzae***	0.05 (0.01–0.09)	0.03 (−0.02–0.08)	0.01 (−0.08–0.10)	0.03 (−0.01–0.07)	0.03 (−0.02–0.08)	0.00 (−0.08–0.09)
**Time between visits (in years)**	0.01 (0–0.02)	0.01 (0–0.02)	0.01 (−0.01–0.02)	0.01 (0–0.02)	0.01 (0–0.02)	0 (−0.01–0.02)
**FEV_1_ at baseline**	−0.06 (−0.09–−0.04)	−0.07 (−0.10–−0.05)	−0.03 (−0.06–0.01)	NA	NA	NA

Results are presented as β-coefficients for univariate or adjusted models, with 95% confidence intervals. TEM: transmission electron microscopy; ODA: outer dynein arm defect; IDA: inner dynein arm defect, MTD: microtubular disarrangement; *P. aeruginosa*: *Pseudomonas aeruginosa*; *H. influenzae*: *Haemophilus influenzae*; NA: not applicable. ^#^: models were adjusted for FEV_1_ values at baseline.

### Long-term change in FEV_1_ z-score

We found a mean annual absolute decrease of 0.06 (95% CI −0.14–0.00) and 0.11 (95% CI −0.20–−0.01) in FEV_1_ z-scores in the unadjusted and adjusted models, respectively (supplementary figure S3). In the adjusted models, there was a high similarity of measurements within individual patients (ICC 0.84) and the variance was mainly explained by differences between patients (ICC 0.81) rather than countries (ICC 0.03). The relative annual decrease was 0.18% (95% CI −1.90–2.27%) in an unadjusted model.

## Discussion

In this large multicentre cohort with >250 PCD patients from 12 countries, we found high intra-individual variability of FEV_1_ z-scores in patients in stable state. We found that relative changes in FEV_1_ >25% compared to the previous measurement should be considered above test variability, and therefore physiologically relevant in PCD patients. Since relative changes are related to the patients’ previous measurements, previous measurements will influence the magnitude of this change in absolute terms, *e.g.* a relative decrease of 25% in a patient with FEV_1_ 40% predicted would mean an observed FEV_1_ of 30% pred, while for a patient with preserved lung function (100% pred) this would translate as greater drop to 75% pred. Previous studies in healthy children have found a decrease in the correlation between repeated measurements when there is an increase in time between measurements; however, we found that test variability remained high even when restricted to patients who had more than three measurements and when measurements were taken <4 months apart [27–29]. Between-measurement variability was slightly higher in children (26.9%) than adults (20.5%), but remained considerably high in both groups. For comparison, year-to-year changes in FEV_1_ >15% over 1 year and relative change of 12% from baseline are considered to be physiologically meaningful in healthy adults, while in children an absolute change within ±1.3 z-scores is considered meaningful [3, 4, 30]. In cystic fibrosis (CF), absolute changes in FEV_1_ >10% compared to baseline are considered meaningful [31].

Individual relative changes of FEV_1_ ranged from 1% to 81% compared to the previous measurement. This high variability could be due to differences in mucus secretions in the larger airways, which in turn may depend on when the measurements occurred, *e.g.* before or after physiotherapy. When assessing the potential determinants of individual lung function variability, we found that patients with central pair defect and lower FEV_1_ values at baseline had decreased variability, while those with ODA defects had higher variability. However, these factors could only account for a relatively small proportion of the total observed variability, and therefore larger studies are needed to confirm these findings and to further explore the potential reasons for the differences observed. Additionally, our analyses were based on small numbers of participants per TEM defect and therefore we could not draw conclusions on whether different ultrastructural defects have any effect on FEV_1_ variability. The impact of high individual variability on disease severity and long-term survival has been explored in CF, with some reports of an association with worse lung function decline [32].

The CR quantifies the absolute reliability measurement error (including both random and systematic errors) and sets the boundary of the minimally detectable true change that can be measured by an instrument. Since it is in the same unit as the measurement obtained (*i.e.* FEV_1_ z-scores, in this case), the CR is adjusted for sex, age, height and ethnicity [24]. We found that changes in FEV_1_ z-score <1.88 between measurements might simply be due to test variability, *i.e.* changes between −0.88 and 2.88 FEV_1_ z-scores. This large absolute reliability measurement error suggests that spirometry would only be able to reliably detect large changes in FEV_1_ z-scores and would potentially miss early signs of lung function deterioration.

Spirometry is the most commonly used test to assess lung function due to its wide availability in clinical settings, affordability of equipment (even in resource-limited settings), ease of application, standardisation in measurements and reporting, and interpretability of results. Importantly, FEV_1_ is one of the proposed core outcomes set measures that should be consistently measured and reported in PCD studies, particularly in randomised controlled trials (RCTs) and prospective cohorts [33]. However, spirometric indices might not be sufficiently sensitive to monitor disease in PCD, as patients with structural and functional lung impairment can have FEV_1_ values within the normal range [34]. In RCTs investigating the efficacy of new drugs in PCD, using FEV_1_ as the main end-point could lead to physiologically meaningful changes not being detected due to high test variability [10, 11]. Furthermore, studies in patients with CF have shown that day-to-day variations in FEV_1_ from home spirometry are common and intra-individual variations can be as high as 16.3% [34–39]. Our results, which show extremely high variability of FEV_1_ in PCD, should raise concerns for the use of FEV_1_ as an outcome parameter in RCTs, especially if used as the primary outcome.

Various studies investigated the variability of lung clearance index derived from MBW in children with CF. Studies reported a coefficient of variation of 7.4–8.2%, with a ULN between visits ranging from 19% to 24% [40, 41]. Within-individual variability of LCI measurements was 10%, compared to 16% for FEV_1_ % predicted [42]. Large prospective cohorts comparing lung function in PCD patients, measured by spirometry and MBW, with structural and functional abnormalities, detected through high-resolution computed tomography and magnetic resonance imaging, are still lacking [43].

Our study is the first to prospectively assess intra-individual variability of FEV_1_ from spirometry in stable PCD patients, performed by clinicians during routine clinical appointments. Our findings enable the interpretation of FEV_1_ in routine clinical surveillance of lung disease in PCD and highlight the potential limitations of using FEV_1_ values to guide therapeutic decisions, both clinically and as an end-point for RCTs. Findings can be generalised to the wider PCD community, as we included both children (aged >5 years) and adults from 19 PCD centres in 12 countries, with different clinical and genetic backgrounds and lung disease severity. The study's long follow-up time allowed us to explore whether having more than two lung function measures reduced FEV_1_ variability. We prospectively collected data using a standard form, and lung function was measured and reported following a pre-defined study standard operation procedure. Additionally, we checked the quality of spirometry measurements obtained at different centres by checking the volume–time and flow–volume curves on test reports for the first five consecutive patients recruited into the study for each centre. We also controlled for data quality by inspecting individual trajectories in FEV_1_ z-scores stratified by country and by applying multilevel models to adjust for clustering by country so that potential differences in spirometry equipment and how measures were obtained could be taken into account.

However, results must be interpreted considering study limitations. We were unable to apply the criteria based on the expert consensus statement to define pulmonary exacerbation in PCD, as we did not collect all the necessary data, since the consensus was published while our study was ongoing [44]. The consensus does not include changes in FEV_1_ as a criterion to define exacerbation, whereas in our study, clinicians managing the patients might have considered a drop in FEV_1_ as a sign of pulmonary exacerbation. Results based on the consensus statement may have been different. Only 45% of patients included in our study had a confirmed diagnosis of PCD based on genetic testing. This study contains data from 2017 to 2019, when genetic testing was less routinely done as part of the diagnostic work-up, particularly in centres with limited resources where a diagnosis was often based on TEM hallmark defects.

### Conclusions

Our findings suggest that changes >25% in intra-individual measurements of FEV_1_ derived from spirometry in stable PCD patients should be considered beyond the expected test variability. However, it is important to consider that all test results, including FEV_1_, must be interpreted by clinicians with knowledge of the individual patient's clinical condition, history and disease status, as statistically insignificant (or significant) changes can be meaningful (or meaningless) depending on the individual's clinical situation, particularly as we have shown a large range of individual FEV_1_ relative change in our population.

This study provides important information for ongoing and future RCTs, particularly those focused on developing and testing new drugs designed to improve lung function and/or impede progression of lung disease in PCD, as our findings suggest that, when used in isolation, FEV_1_ is not a sensitive test for monitoring lung health in PCD [12]. More sensitive markers of lung function in PCD, such as LCI derived from MBW, should be considered when selecting outcome measures for future pulmonary intervention studies.

## Supplementary material

**Please note:** supplementary material is not edited by the Editorial Office, and is uploaded as it has been supplied by the author. Supplementary material 01115-2024.SUPPLEMENT
