# Supplementary material for: Natural variability of lung function in primary ciliary dyskinesia: longitudinal analysis from the PROVALF-PCD cohort
Source: ERJ Open Res. 2025 Jun 23;11(3):01115-2024. doi: 10.1183/23120541.01115-2024 (PMC12183702; doi:10.1183/23120541.01115-2024)
Supplement: Supplementary file 1 [file 01115-2024.SUPPLEMENT.pdf]

Supplementary

Spirometry measurements and quality control

In order to obtain an acceptable blow, the following conditions had to be met: a) satisfactory start of expiration (i.e. no excessive hesitation or false start back extrapolated volume or back extrapolated volume >5% of forced vital capacity (FVC)), b) no cough during the first second of the manoeuvre or any cough after that in which, in the operator's judgement, interferes with obtaining accurate results, c) no early termination of expiration, d) no cessation of airflow due to Valsalva manoeuvre or hesitation, e) no leak, f) no obstruction of the mouthpiece, and g) no extra breath was taken during the manoeuvre. A minimal of 3 manoeuvres with 1 minute interval between blows was required. After 3 acceptable spirograms were obtained, the 2 largest values of FVC and of forced expiratory volume in 1s (FEV_1) should be within 0.15 litres of each other to meet the repeatability criteria. The best measurements obtained that met the acceptable blow and repeatability criteria were recorded in the study CRF.

Test operators were instructed to visually inspect both flow-volume and volume-time displays during each manoeuvre before proceeding with subsequent manoeuvres to ensure measurements were of good quality. Centres were requested to communicate any changes of spirometry equipment that occurred during the study to the coordinating centre. They were instructed to upload anonymised copies of spirometry reports for the first five consecutive participants recruited into the study, which were reviewed by the coordinating centre.

Statistical analyses

Relative change in FEV_1 between consecutive visits in stable participants was calculated as follows:

$$(FEV1_{i+1} - FEV1_i)/FEV1_i * 100\%$$

, where FEV_1 is the FEV_1 raw value (in litres) and i represents the clinic appointment at which lung function was measured. To calculate the upper limit of normal (ULN), we log-transformed FEV_1 by the natural logarithm, and then fitted an unadjusted multilevel model with random intercept at patient level. Based on this model, we calculated the ULN for relative change (95% quantile of normal distribution) as below:

$$\text{ULN} = e^{\sqrt{2}*1.64*\sigma} - 1$$

, where σ is the residual SD derived from the multilevel model.

To determine limits for each individual, we calculated ULN of relative changes of FEV_1 between measurements for each patient in stable condition. The formulae used is similar to the one shown above; however, σ in this instance represents the SD of log transformed FEV_1 values.

We also calculated the coefficient of repeatability (CR) using the following formula:

$$CR = 1.96 \times \sqrt{2} \times \sigma_w$$

, where σ_w is the within subject SD.

Results for patients experiencing an episode of pulmonary exacerbation

The mean absolute difference of FEV_1 z-scores between visits in patients that were stable in the first clinical appointment but had an exacerbation in the subsequent appointment was -0.23 (SD 0.56). The ULN for patients experiencing an exacerbation was 29%, marginally higher than the ULN observed for patients in stable state (i.e. 25%).

Results for FVC as outcome

Results for FVC were similar to those for FEV_1. The upper limit of normal (ULN) between two consecutive measurements of FVC obtained 6 months apart or less was 25.2% in stable patients (versus 25% for FEV1) and the coefficient of repeatability (CR) was 2.09 (versus 1.88 for FEV_1).

Table S1. Number of participants with primary ciliary dyskinesia (PCD) included in the study and number of visits per patient, stratified by country.

**Country**	**n patients (%)**	**Median number of visits per patient (range)**
England	26 (10.3%)	4.5 (2 to 8)
Australia	6 (2.4%)	3 (3 to 4)
Italy	34 (13.5%)	4 (2 to 6)
Denmark	18 (7.1%)	3.5 (2 to 7)
Germany	29 (11.5%)	4 (2 to 10)
Cyprus	15 (6.0%)	6 (3 to 8)
France	15 (6.0%)	4 (2 to 6)
Belgium	22 (8.7%)	4 (3 to 9)
Portugal	6 (2.4%)	4.5 (3 to 7)
Turkey	38 (15.1%)	5 (2 to 8)
Czech Republic	17 (6.7%)	5 (3 to 9)
Spain	26 (10.3%)	5 (3 to 8)
**Total**	**252 (100%)**	**3 (2 to 4)**

Table S2. Details of covariates adjusted for in multilevel model of longitudinal changes in forced expiratory volume in 1s (FEV_1) z-score in patients with primary ciliary dyskinesia (PCD).

**Covariates**	**n visits (%)**
**Presence of respiratory pathogens**	737 (62.6)
**Health status (compared to baseline)**	
Very well	200 (17.0)
Well	655 (55.6)
Somewhat well	220 (18.7)
Ill	70 (5.9)
Very ill	0 (0)
**Courses of antibiotics since last visit (median, IQR)**	0 (0 to 1)
**Use of antibiotic prophylaxis**	415
**Use of inhaled corticosteroid**	449

Figure S1. Forced expiratory volume in 1s (FEV_1) z-score individual trajectories for participants in stable state, stratified by country.

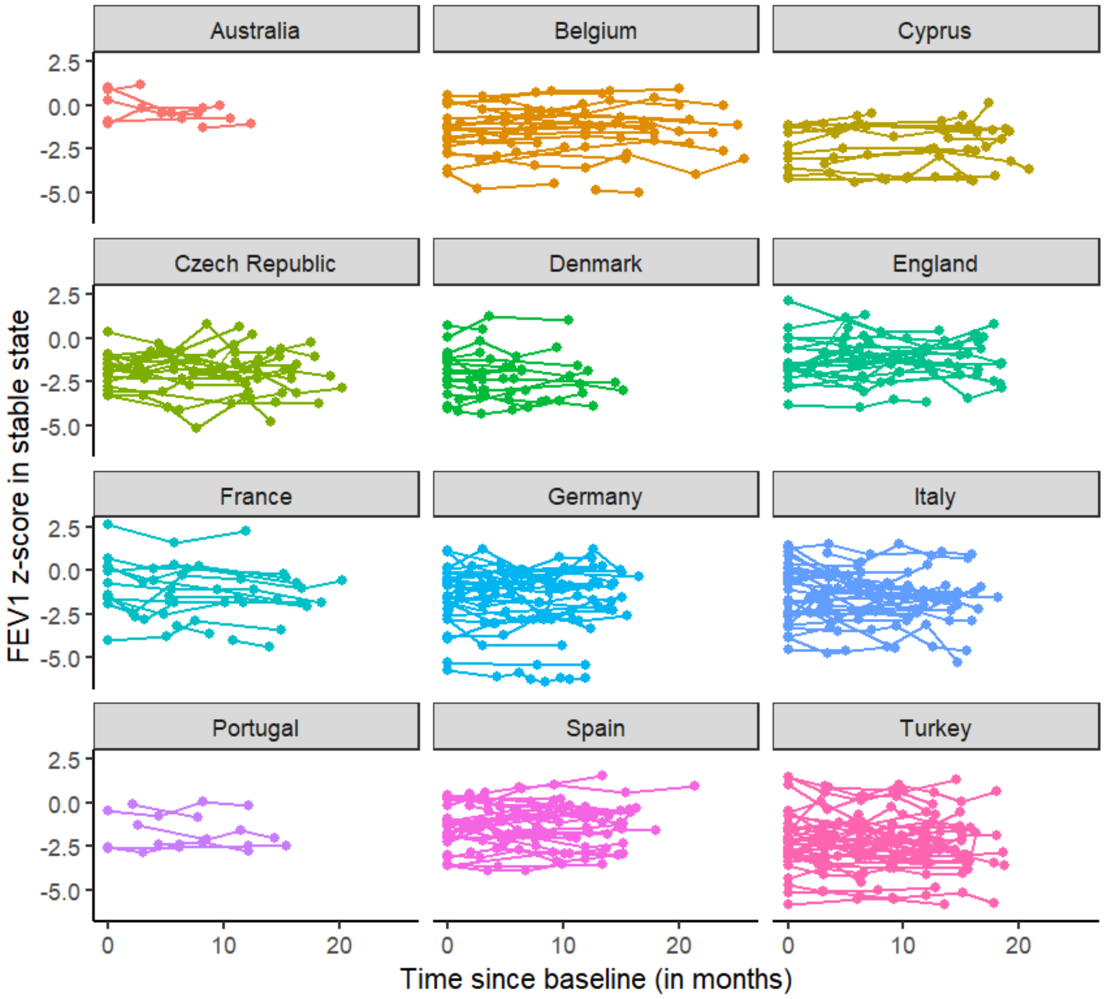

Zhang et al. Natural variability of lung function in primary ciliary dyskinesia: longitudinal analysis from the PROVALF-PCD cohort.

Figure S2. Venn diagram showing the numbers of abnormal diagnostic tests for each diagnostic test and for the combinations of diagnostic tests.

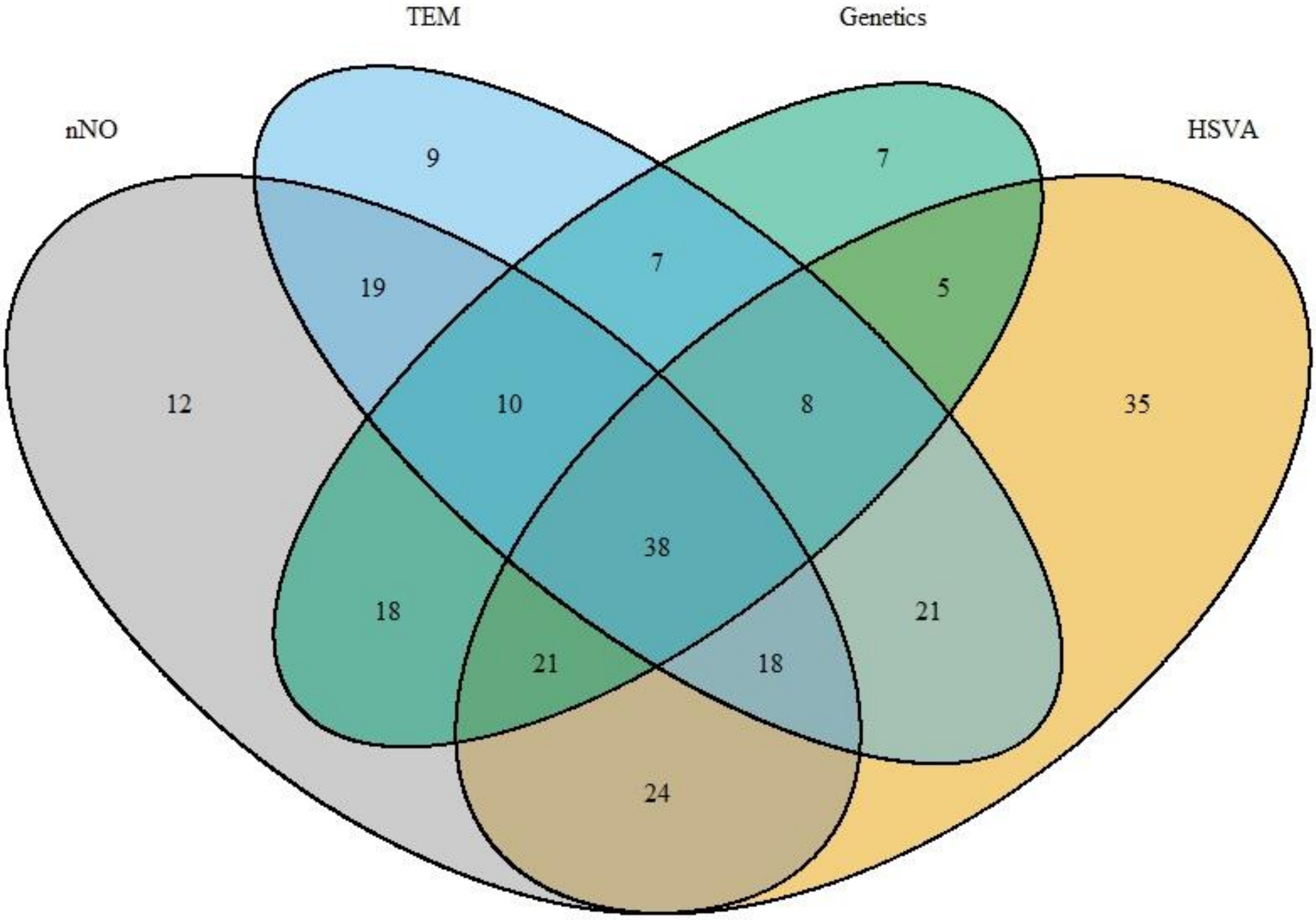

Zhang et al. Natural variability of lung function in primary ciliary dyskinesia: longitudinal analysis from the PROVALF-PCD cohort.

Figure S3. Coefficient estimates for forced expiratory volume in 1s (FEV_1) z-scores in adjusted multilevel model of rate of lung function decline in patients with primary ciliary dyskinesia (PCD).

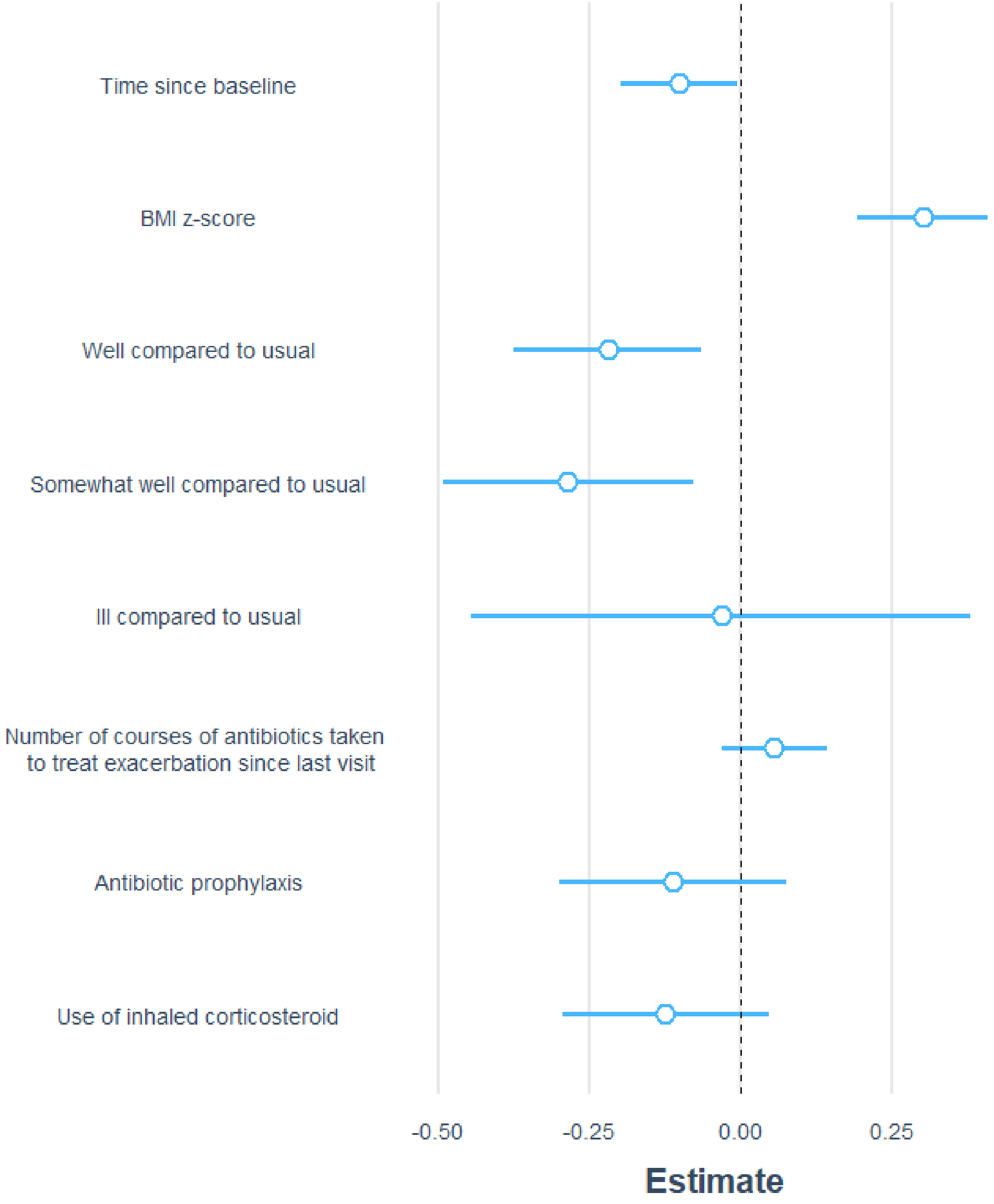

Model was adjusted for time since baseline, BMI z-score, current health compared to usual (reference category: very well compared to usual), number of courses of antibiotics, use of antibiotic prophylaxis, and use of inhaled corticosteroids. Estimates are represented by the circles, and the 95% confidence intervals by the whiskers.
